# Excess Dietary Manganese Impairs Iron Nutrition via Modulating Duodenal Transporters in Weaned Pigs

**DOI:** 10.3390/vetsci12121118

**Published:** 2025-11-25

**Authors:** Maolian Wei, Yuhuai Xie, Ruonan Yan, Jiming Liu, Wenli Tang, Yuming Zhan, Li Qiang, Zhiqiang Yang, Lingling Gong, Shuzhen Jiang, Weiren Yang

**Affiliations:** 1 Shandong Provincial Key Laboratory of Animal Biotechnology and Disease Control and Prevention, College of Animal Science and Technology, Shandong Agricultural University, Tai’an 271018, China; wmlsyol@163.com (M.W.); szjiang@sdau.edu.cn (S.J.); 2 Shandong Provincial Key Laboratory of Quality Safety Monitoring for Animal Products and Veterinary Drug Innovation, Shandong Center for Quality Control of Feed and Veterinary Drug, Jinan 250100, China; 13805415908@163.com (J.L.); 13905310419@163.com (W.T.); 13589018038@163.com (Y.Z.); sdslsql@163.com (L.Q.); 13969191026@163.com (Z.Y.); 13869133827@163.com (L.G.); 3 College of Veterinary Medicine, Shandong Agricultural University, Tai’an 271018, China; yhxie@sdau.edu.cn; 4 School of Medicine, Huanghe Science and Technology College, Zhengzhou 450061, China; 5 Tai’an Tumor Hospital, Tai’an Third People’s Hospital, Tai’an 271000, China; yrn2024@163.com

**Keywords:** manganese, iron, divalent metal transporter 1, ferroportin 1, weaned pigs

## Abstract

Despite manganese’s essential role as a cofactor for multiple enzymes, its potential to disrupt iron homeostasis when supplemented in excess remains a critical knowledge gap in swine nutrition. This study evaluated the effects of Mn (manganese)-supplemented diets on growth, hematology, mineral accumulation, digestibility, and intestinal iron transporter expression in weaned pigs. A total of 128 crossbred pigs (Duroc × Landrace × Largewhite) with an average body weight of 9.82 ± 0.15 kg were randomly allotted to four dietary treatments comprising a basal diet supplemented with 0, 20, 40, or 80 mg MnSO_4_ per kg diet for 28 days. Blood samples were collected from 16 weaned pigs (1 pig per pen, 4 per treatment), after which animals were euthanized for tissue sampling. No differences were observed in growth performance. However, Mn concentrations in serum, liver, heart, kidney, pancreas, and metatarsal bones increased both linearly and quadratically with increasing dietary Mn (*p* < 0.05), and Fe (iron) concentrations in serum, hemoglobin, liver, and metatarsal bone decreased (*p* < 0.05). Apparent digestibility data further revealed that Mn absorption peaked at 20 mg/kg, while Fe digestibility declined linearly with increasing Mn levels. Moreover, duodenal *DMT1* (divalent metal transporter 1) mRNA expression was decreased, whereas *FPN1* (ferroportin 1) was upregulated. These findings demonstrate that excessive Mn supplementation impairs dietary Fe absorption and homeostasis in weaned pigs, suggesting that the antagonism occurs at both the transcriptional and intestinal absorption levels, providing critical insights for dietary formulation in swine.

## Simple Summary

Manganese is a necessary mineral for animal health, but we wanted to understand what happens when pigs get too much of it in their diet, particularly how it affects their iron levels. We fed weaned pigs with different amounts of manganese and then checked their blood, organs, and bones. We found that high levels of dietary manganese made it harder for the pigs to absorb and use iron, leading to lower iron levels in their bodies. This happened because manganese changed the activity of special gates in the gut that control iron entry into the body. Importantly, the pigs’ growth was not affected, meaning this iron problem can occur even when the animals appear healthy. Our research helps pig farmers formulate safer and more effective diets by showing that excessive manganese can cause a hidden iron deficiency, and that a moderate amount is sufficient for the animals’ needs.

## 1. Introduction

In monogastric animals, manganese (Mn) and iron (Fe) are known to interact antagonistically during intestinal absorption, yet the molecular basis of this interaction in pigs remains elusive. As an essential micronutrient, Mn serves as a critical cofactor for numerous enzymes involved in antioxidant defense, bone development, and metabolism [1,2,3]. However, establishing its optimal dietary level for weaned pigs must account for this potential interference with Fe nutrition, an aspect that is currently poorly defined.

Excessive dietary Mn has been reported to suppress growth in rats [4,5] and chicks [6]. However, studies in modern swine genetics indicate that growth performance is an insensitive criterion for manganese requirements. Most recently, Kerkaert et al. concluded that there is little benefit in growth performance by feeding more than 8 mg/kg of added Mn to growing-finishing pigs [7]. Supporting this, Pallauf et al. demonstrated that Mn deficiency or excess primarily affects tissue Mn deposition and enzyme activity long before alterations in growth performance become apparent, highlighting the need for more sensitive metabolic indices [8]. Even at exceedingly high inclusion levels reaching 320–350 ppm, Apple et al. observed no adverse effects on the average daily gain of pigs [9]. This collective evidence from contemporary research firmly establishes that this apparent higher tolerance further suggests that growth performance alone is an insufficient criterion for determining Mn requirements in this species, necessitating a focus on more sensitive metabolic indices.

Specifically, there is a notable lack of information regarding the effect of dietary Mn on the expression of intestinal Fe transporters in pigs. Moreover, the potential Mn-Fe interaction at the site of absorption and its underlying mechanism have not been systematically investigated in previous swine studies. Therefore, the objectives of this study were to (1) quantify the dose–response effect of dietary Mn on tissue Mn and Fe accumulation and their apparent digestibility in weaned pigs, (2) investigate its impact on the gene expression of key Fe transporters, and (3) establish an appropriate dietary Mn level for weaned pigs based on a comprehensive assessment of mineral status, digestibility, and transporter dynamics.

## 2. Materials and Methods

### 2.1. Animals, Diets, and Housing

The animal care and use protocol was reviewed and approved by the Animal Nutrition Research Institute of Shandong Agricultural University (Approval Code: SDAUA-2024-050). All animal handling and experimental procedures followed the committee’s established guidelines for the humane care and use of laboratory animals.

A total of 128 weaned pigs (Duroc × Landrace × Largewhite), weaned at 28 days of age with an average body weight of 9.82 ± 0.15 kg were used in a 28-day study. All pigs were confirmed to be healthy by a veterinarian at the start of the experiment, based on visual inspection for signs of illness (e.g., lethargy, coughing, diarrhea) and normal feeding behavior. The pigs had received a standard vaccination program prior to the trial, which included vaccinations against classical swine fever, porcine circovirus, and porcine reproductive and respiratory syndrome. At the start of the experiment, pigs were blocked on the basis of initial body weight, while equalizing ancestry and gender across treatments (4 barrows and 4 females), into four dietary treatments in a randomized complete block design of 16 pens (8 pigs/pen, 4 pens/treatment). All pigs received a standard prophylactic intramuscular injection of 200 mg iron as iron dextran on day 3 postpartum to prevent neonatal iron deficiency anemia, ensuring adequate iron status at the initiation of the experiment. The dietary treatments consisted of a basal control diet (Table 1) and the basal diet supplemented 20, 40, or 80 mg/kg Mn from manganese sulfate (MnSO_4_; reagent grade, >99% purity, containing 31.8% Mn; Scipro Biological Technology Co., Ltd., Chengdu, China). The basal diet was formulated to meet or exceed the nutrient requirements for weaned pigs as recommended by the NRC (1998) [10] and was confirmed by flame atomic absorption spectrometry to contain 39.22 mg/kg Mn and 125 mg/kg Fe.

The pigs were housed in a temperature-controlled nursery room at Jinzhuyuan Farm (Yinan, Shandong, China). The temperature of the barn was set between 26 and 29 °C, and the mean relative humidity was maintained at approximately 65%. Each pen (1.8 m × 2 m) had a sloped concrete floor with a drainage hole and contained a two-hole, stainless steel feeder and a nipple drinker.

### 2.2. Evaluation of Performance and Sample Collection and Processing

The feed experiment lasted 28 d. Body weights were measured weekly and at the end of the experiment, and feed intakes and refusals of the pigs were recorded daily. Average daily gain, average daily feed intake (ADFI) and FE (ADG/ADFI) were calculated over the 28-day feeding period.

At the end of the experimental period, four pigs were randomly selected from each treatment (1 pig/pen), and blood samples were collected from the selected pigs via the vena cava duct on a morning after being fasted for 12 h. For hematological testing, 3.0 mL samples were collected in test tubes containing an anticoagulant (EDTAK_2_, Yunmeida Biotechnology Co., Ltd., Harbin, Heilongjiang Province, China), and 10.0 mL samples were collected in test tubes (Kangweishi Medical Technology Co., Ltd., Shijiazhuang, Hebei Province, China) without anticoagulant for biochemical analyses. Serum samples were obtained after centrifugation of clotted blood at 3000× *g* for 15 min, and the serum was stored in 1.5 mL Eppendorf tubes (Eppendorf SE, Hamburg, Germany) at −20 °C until analysis.

After the collection of blood samples, the pigs were immediately euthanized with an intravenous injection of 10% sodium pentobarbital at a dose of 150 mg per kg body weight. At this time, the duodena were removed from the pigs. A segment from the proximal duodenum, taken from a region 5 cm distal to the pylorus, was washed with 0.9% saline to remove any digesta. Approximately 15.0 g of tissue was immediately frozen in liquid nitrogen and stored in a freezer at −80 °C for analyses of divalent metal transporter 1 (*DMT1*) and ferroportin 1 (*FPN1*) expression. Samples (~200 g) from the right lobe of the liver, the whole right kidney, pancreas, and heart were quickly removed, washed briefly in cold phosphate buffer, and stored at −80 °C until analysis, as well. Finally, the left legs of the pigs were excised and stored at −20 °C in individual heat-sealed polyethylene bags until analysis.

### 2.3. Mineral Analyses

The Mn and Fe concentrations of both diets and tissues were analyzed using flame atomic absorption spectrophotometry (model AA2600; Beijing Chaoyang Foreign Analysis Instrument Co., Ltd., Beijing, China). Metatarsal bones from the left legs were boiled in deionized water for 10 min, cleaned of soft tissue, dried at 105 °C for 12 h, and charred in a muffle furnace at 550 °C for 16 h. Approximately 0.2 g bone ash, 3.0 g heart, 2.0 g liver, 2.0 g kidney, and 2.0 g pancreas were digested with a 10:1 (*v*/*v*) mixture of HNO_3_ (nitric acid) and HClO_4_ (perchloric acid) at 200 °C in a 50 mL calibrated flask until clear and condensed to ~0.2 mL. Diet samples were similarly processed. The clear digests were diluted with deionized water (1:50) and analyzed against a series of certified standard solutions to establish a calibration curve (R^2^ > 0.999). To ensure accuracy, a standard reference material (Porcine Liver, GBW10051) was included in each batch of analyses, with recovery rates ranging from 95% to 105%. Each sample was analyzed in quadruplicate. All analyses were performed in quadruplicate, and the mineral concentrations of the diets, fresh tissues (liver, heart, kidney, and pancreas), and bone were reported on an as-fed, fresh, and ash basis, respectively. Analysis of the experimental diets verified the intended Mn supplementation gradients (analyzed values: 0, 20, 40, 80 mg/kg added groups contained 39.2, 58.5, 78.8, and 118.0 mg/kg Mn, respectively) and confirmed consistent iron levels across all diets (approximately 125 mg/kg). A different sample preparation approach was employed for serum. Serum samples were diluted 1:5 with 0.1% nitric acid, vortexed thoroughly, and centrifuged at 10,000× *g* for 10 min. The clear supernatant was then directly aspirated into the AAS for measurement of Mn and Fe concentrations.

### 2.4. Determination of Apparent Digestibility

The apparent digestibility of manganese (Mn) and iron (Fe) was determined using a total fecal collection method from days 11 to 16 of the experiment. Following collection, feces from each pen were thoroughly mixed daily. A representative sample, equivalent to 10% of the total daily fecal output, was taken. At the end of the 5-day collection period, all daily samples from the same pen were pooled and mixed thoroughly to form a composite sample. About 500 g portion of this fresh composite sample was obtained by quartering. This sample was then analyzed for initial moisture content, crushed with a mortar, sieved through a 40-mesh screen, and stored for subsequent analysis of Mn and Fe concentrations.

The Mn and Fe concentrations in the diets and the prepared fecal samples were determined by flame atomic absorption spectrophotometry, as described in Section 2.3. The apparent digestibility of each mineral was calculated for each pen (the experimental unit) using the following formula: Apparent Digestibility (%) = [(Intake − Fecal Output)/Intake] × 100; Where Intake (mg/d) = Daily feed intake (g, as-fed basis) × Dietary mineral concentration (mg/g). Fecal Output (mg/d) = Daily fecal output (g, dry matter basis) × Fecal mineral concentration (mg/g). The apparent digestibility was determined using the total collection method without an indigestible marker. While this method effectively captures the relative differences between treatments, it should be noted that absolute values may include endogenous mineral losses.

### 2.5. RNA Extraction, Reverse Transcription, and Real-Time PCR

Total RNA was isolated from duodenal tissue using TRIzol reagent (Invitrogen, Shanghai, China). The concentration and purity of the RNA were determined using a NanoDrop spectrophotometer (Thermo Scientific, Sunnyvale, CA, USA), with all samples having A260/A280 ratios between 1.8 and 2.0, indicating pure RNA without significant protein or solvent contamination. RNA integrity (RIN) values were more than 8.0 and confirmed by 1.5% agarose gel electrophoresis, showing clear 18S and 28S rRNA bands. cDNA was generated using a reverse transcription kit (Invitrogen, Shanghai, China), and a no-reverse transcription (no-RT) control was included for each sample to confirm the absence of genomic DNA contamination according to the manufacturer’s protocol. The mRNA expression levels were detected using real-time PCR (GeneAmp PCR System; Applied Biosystems, Foster City, CA, USA) with a reaction volume of 20 μL. The following cycling conditions were used for all amplifications: 2 min at 95 °C (pre-incubation), 10 s at 95 °C (denaturation), 30 s at 60 °C (annealing), and 30 s at 72 °C (extension), and a final melting curve analysis. A dissociation stage was added to the PCR procedure to ensure the specific amplification of each primer pair. In addition, a standard curve was generated from a dilution series of cDNA samples in order to determine the amplification efficiency of each primer pair. The relative expression levels of the target genes were measured using qRT-PCR, calculated using the 2^−ΔΔCt^ method, and normalized using GAPDH (glyceraldehyde-3-phosphate dehydrogenase) as an endogenous control gene. The stability of GAPDH expression was validated across all treatment groups, with Ct values showing minimal variation (CV < 0.5%). The amplification efficiency for all primer pairs, determined from the standard curve, was between 95% and 105% (R^2^ > 0.999). Each qPCR reaction was performed in triplicate (technical replicates). Primers were synthesized by Invitrogen (Shanghai, China, Table 2).

### 2.6. Determination of Hematological Parameters

Hematological analysis was performed on fresh whole-blood samples collected in EDTA-anticoagulated vacuum tubes (EDTAK_2_, Yunmeida Biotechnology Co., Ltd., Harbin, Heilongjiang Province, China). Hemoglobin and hematocrit were determined using an automatic blood analyzer (KX-21 model; SYSMEX Pacific-Asia Co., Kobe, Japan) according to the manufacturer’s standard operating procedures and the instrument’s integrated measurement principles. To ensure the accuracy and precision of all results, the analyzer was calibrated prior to the study using manufacturer-provided calibrators. Furthermore, quality control was performed daily using commercial three-level control materials (covering low, normal, and high ranges) before any patient samples were analyzed.

### 2.7. Statistical Analyses and Calculation

All data were subject to analysis of variance using SAS (version 9.1; SAS Institute, Cary, NC, USA). In addition, linear and quadratic responses to increasing levels of dietary Mn were determined, and differences among treatments were examined using Duncan’s multiple range test. In all analyses, significant differences were declared at *p* < 0.05, and tendencies were defined at 0.05 < *p* > 0.1.

## 3. Results

Throughout the experimental period, the weaned pigs were generally healthy as monitored daily by visual inspection for signs of illness and normal feeding behavior; however, one mortality occurred, without a known cause, and was therefore excluded from the data calculation and statistical analysis.

### 3.1. Growth Performance

Increasing dietary Mn levels did not affect ADG, ADFI, or FE (*p* > 0.10; Table 3). However, the highest ADG and the lowest FE were both observed in the group supplemented with 20 mg Mn/kg.

### 3.2. Hemoglobin, Hematocrit, and Serum Mn and Fe

Supplementation of Mn from 20 to 80 mg/kg did not affect hematocrit value (*p* > 0.05; Table 4). However, the pigs fed diets supplemented with 80 mg/kg Mn exhibited higher serum Mn (*p* < 0.05) and lower Fe and hemoglobin concentrations (*p* < 0.05) than pigs fed other diets. Serum Mn levels were higher linearly and quadratically with increasing dietary Mn, and both blood hemoglobin and serum Fe levels were lower linearly and quadratically with increasing dietary Mn.

### 3.3. Tissue Mn and Fe Content

**Figure 1:**
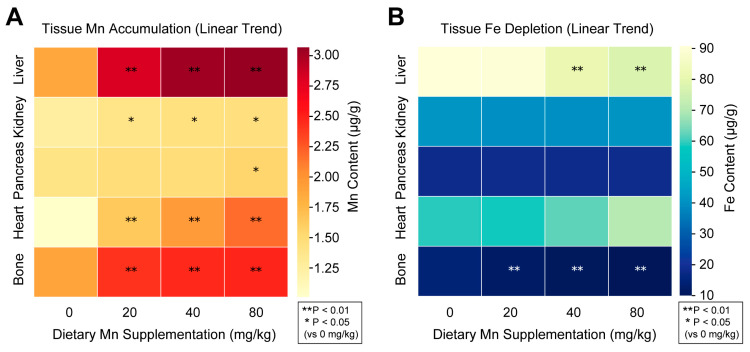
**Tissue manganese (Mn) accumulation and iron (Fe) depletion in weaned pigs fed increasing dietary Mn (0, 20, 40, 80 mg/kg diet).** (**A**) Mn heatmap: Color gradient (yellow to red) shows Mn concentrations (μg/g wet tissue), with warmer colors indicating higher accumulation. Asterisks denote significant linear increases vs. 0 mg/kg control (** *p* < 0.01, * *p* < 0.05). (**B**) Fe heatmap: Color gradient (light to dark blue) shows Fe concentrations, with darker shades indicating depletion. Asterisks mark significant linear decreases (** *p* < 0.01). Tissue samples (*n* = 4 per treatment) were collected at the end of the 28-day trial, and mineral concentrations were determined by flame atomic absorption spectrometry after acid digestion. Data are presented on a fresh tissue weight basis.

The concentrations of Mn in liver, kidney, pancreas, heart, and bone tissues were higher (Linear *p* < 0.05; Quadratic *p* < 0.05) as Mn supplementation increased from 20 to 80 mg/kg diet (Table 5 and Figure 1). In addition, the Fe concentrations of liver and bone tissues were lower when pigs were fed 40 mg/kg and 20 mg/kg Mn and were lower linearly and quadratically with increasing Mn supplementation. However, there were no significant differences in the Fe concentration in kidney, pancreas, and heart tissues of pigs that were fed different levels of Mn.

### 3.4. The Apparent Digestibility of Manganese and Iron

**Figure 2:**
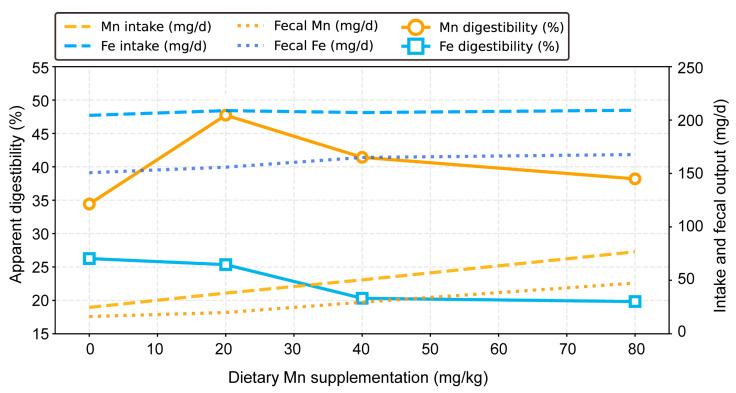
**Dose-dependent effects of dietary manganese supplementation on manganese and iron apparent digestibility in weaned pigs.** (1Manganese (circle, orange) and iron (square, blue) apparent digestibility (%, solid lines). Mn digestibility peaked at 20 mg/kg (47.74%), showing a quadratic trend (*p* = 0.032), whereas Fe digestibility decreased linearly (*p* < 0.05) with increasing Mn supplementation. (2) Corresponding daily intake (dashed lines) and fecal excretion (dotted lines) patterns. See Table 6 for complete statistical results and significance letters. Apparent digestibility was determined via total fecal collection (*n* = 4 pens per treatment). Each data point represents the mean value for a treatment group.

Dietary Mn supplementation significantly affected Mn and Fe apparent digestibility (Table 6, Figure 2). Mn digestibility peaked at 47.74% with 20 mg/kg supplementation, then declined with higher levels (Quadratic *p* = 0.071). In contrast, Fe digestibility showed a significant linear decrease (*p* < 0.01), dropping to 19.82% at 80 mg/kg Mn. These findings demonstrate that optimal Mn absorption occurs at moderate supplementation, while excess Mn directly impairs Fe uptake at the intestinal level.

### 3.5. Expression of Fe Transporter Genes

**Figure 3:**
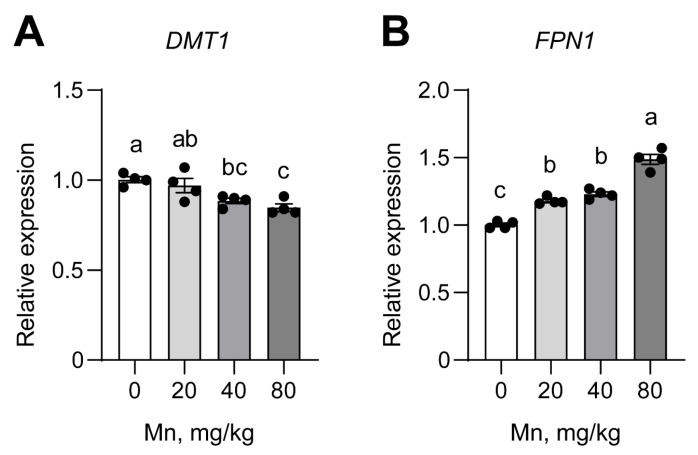
Effect of dietary Mn on the relative mRNA expression of *DMT1* (**A**) and *FPN1* (**B**) in the duodena of weaned pigs. Different lowercase letters indicate significant differences (*p* < 0.05). All data are expressed as means ± SEM (*n* = 4). Duodenal mucosal mRNA expression (*n* = 4 per treatment) was analyzed by qRT-PCR. Gene expression levels were normalized to GAPDH and are presented relative to the control group (0 mg/kg Mn).

The *DMT1* mRNA level in the duodenum of weaned pigs was lower with Mn supplementation (*p* < 0.05; Figure 3). Pigs fed 40 mg/kg Mn already exhibited significantly reduced *DMT1* gene expression (*p* < 0.05), and pigs fed 80 mg/kg Mn exhibited the lowest expression. In contrast, the *FPN1* mRNA abundance was significantly higher in pigs fed 20–80 mg/kg Mn (*p* < 0.05).

## 4. Discussion

### 4.1. Effects of Dietary Mn Levels on Growth Performance

The effect of dietary Mn on the growth performance of pigs has been controversial. The results of the present study demonstrated that the growth performance of pigs was not influenced by dietary Mn supplementation, but the ADG and FE of the 40 mg/kg group were numerically higher and lower, respectively, than those of the other groups were. This result is similar to a previous report, in which Mn supplementation of 40 mg/kg increased both ADG and FE [11]; however, when dietary Mn exceeded 55 ppm, the authors failed to note any additional improvements in performance. Svajgr et al. [12] also reported that FE was improved by adding 100 ppm Mn to swine finishing diets, but the improvement was not statistically significant. Conversely, neither Plumlee et al. [13] nor Leibholz et al. [14] observed an effect of dietary Mn on the performance of either weaned pigs or sows, and Svajgr et al. [12] reported that ADG was actually depressed when growing-finishing diets were supplemented with 50 ppm Mn from manganese oxide. However, the differences between studies could, in part, be explained by differences in experimental variables, such as breed, sex, diet type, and weight range of feeding.

### 4.2. Effect of Dietary Mn on Fe and Mn Nutritional Status

Both hemoglobin and hematocrit represent sensitive criteria for evaluating biological responses to iron [15], and serum iron concentration can be used to test for iron deficiency [16]. In this study, pigs received standard iron dextran at birth, ensuring sufficient baseline iron status. The significant reduction in hemoglobin (to 95.25 g/L) and serum iron (to 1.79 μg/mL) in the 80 mg/kg Mn group represents a clear deviation from this adequate baseline. Although the serum iron value of 1.79 μg/mL remains within the established normal range for healthy pigs (1.0–2.1 μg/mL) [17], its significant decline indicates a substantively impaired iron status and provides clear evidence of manganese-induced interference. A whole-blood hemoglobin concentration of 100 g/L is considered adequate, whereas 80 g/L suggests borderline anemia and 70 g/L or less indicates anemia [18]. In fact, when the amount of Mn supplementation reached 80 mg/kg, both hemoglobin and serum Fe were significantly reduced, even though there was no significant change in hematocrit. Although the sample size for blood analysis was *n* = 4 per treatment, the fact that these key parameters reached statistical significance, coupled with the consistent dose-dependent trends observed across tissues, underscores the biological relevance of this finding. Similar results have also been observed in rats [19], as well as in chicks [20].

Crucially, our digestibility data provide direct intestinal-level evidence for this phenomenon: Fe apparent digestibility declined linearly with increasing Mn supplementation, falling to 19.82% at 80 mg/kg Mn. This suppression of Fe absorption at the gut level logically preceded and explains the systemic depletion of Fe stores. It should be noted that digestibility was determined by total collection without an indigestible marker. While this method robustly captured the relative differences between treatments, future studies using markers could provide further validation of absolute values.

During the 28-day feeding experiment, we also found that the Fe concentration was significantly decreased in the livers of pigs that were fed 40 mg/kg Mn-supplemented diets, as was the Fe concentration in bone. Since intestinal Fe absorption regulates Fe homeostasis in the bodies of animals [21,22], these findings provide further evidence that high Mn levels compromise systemic Fe status by impairing its intestinal absorption. This aligns with reports in hens [23], lambs [24], and rats [25]. Physiological events are induced by Fe deprivation and result in four stages of subnormal Fe status [26]. The first stage is depletion, during which the liver, kidney, and spleen Fe stores are reduced, and blood variables related to Fe status are not altered. The onset and duration of the depletion period are determined by the abundance of the initial storage pools, primarily in the liver.

Conversely, the increase in Mn retention observed in the present study suggests that Mn supplementation increased the Mn status of weaned pigs. Similar results were observed in broilers supplemented with 0 to 180 mg/kg Mn [27,28,29]. Notably, Mn apparent digestibility also exhibited a quadratic response, peaking at 47.74% with 20 mg/kg Mn before declining at higher doses. These non-linear absorption kinetics may reflect saturation of Mn uptake pathways or activation of homeostatic exclusion mechanisms at elevated Mn levels.

Collectively, our findings delineate a clear path of Mn-Fe antagonism: high dietary Mn levels directly interfere with iron absorption at the intestinal level, as evidenced by the linear decrease in Fe apparent digestibility. This intestinal competition subsequently leads to systemic iron depletion, marked by reduced serum iron, hemoglobin levels, and hepatic and bone iron stores. The quadratic response in Mn digestibility further suggests a complex, saturable absorption mechanism for Mn itself, which is overwhelmed at higher doses, exacerbating the interference with Fe.

### 4.3. mRNA Abundance of DMT1 and FPN1

The molecular basis for the observed Mn-Fe antagonism at the intestinal level likely involves the key iron transporters, DMT1 [30] and FPN1 [31]. The lower intestinal *DMT1* mRNA level indicated that Mn supplementation was indeed responsible for reduced Fe absorption, which has also been observed in the duodena of chickens [23], ligated duodenal loops of broilers [23,32], and a Caco-2 cell model [33]. The observed reduction in *DMT1* is consistent with a physiological adaptation to limit cellular uptake of excess manganese, as both Fe and Mn are potential substrates for *DMT1* [34]. This view is also supported by in vivo experiments in Belgrade rats, in which a mutation rendering *DMT1* ineffective caused both anemia and reduced Mn concentrations [35].

However, another in vivo study by Garcia et al. [19] showed that *DMT1* expression increased by ~35% in the brains of rat pups nurtured by dams fed with high-Mn diets but decreased in pups nurtured by dams fed with a high-Fe diet. These inconsistent results might be due differences in iron nutrition, since Gunshin et al. [36] reported that duodenal *DMT1* mRNA levels increased considerably in response to Fe depletion. In the present study, for example, the Fe concentrations of hemoglobin, liver, and bone were reduced; however, the Fe levels were still adequate, so no increases in *DMT1* levels were induced.

Similar to DMT1, the *FPN1* protein is expressed in tissues involved in both iron and manganese homeostasis, including the developing and mature reticuloendothelial system and liver, the pregnant uterus, and the basolateral membrane of duodenal epithelial cells [37,38]. Yin et al. [39] implicated *FPN1* as a potential Mn transporter, and Madejczyk and Ballatori [40] provided direct evidence that Mn was a substrate for *FPN1* and that *FPN1* might be a multi-specific metal efflux carrier. In addition, the inducible HEK293T cell model showed that *FPN1* expression reduced Mn-induced toxicity and Mn accumulation, and *FPN1* levels have been shown to increase in response to Mn treatment [41]. Thus, our observation of increased *FPN1* mRNA expression in the duodena of pigs was consistent with in vitro studies using HEK293T cells [39], Caco-2 cell models [33], human epithelial HeLa cells [41], and the duodenal cells of hens [23], which showed that *FPN1* expression was increased by high levels of Mn.

The coordinated downregulation of *DMT1* and upregulation of *FPN1* suggests a physiologically adaptive response: reducing cellular Mn uptake while enhancing its export, thereby mitigating potential manganese overload.

## 5. Conclusions

In conclusion, the results of the present study demonstrate that feeding high concentrations of dietary Mn to pigs affects Fe metabolism, since both liver and bone Fe concentrations were lower in pigs that were fed 40 and 20 mg/kg Mn-supplemented diets, and the reduction in Fe status could be caused by alteration of *DMT1* and *FPN1* expression in the intestine. Therefore, while 20 mg/kg Mn did not impact growth performance and may represent a safe supplementation level from a growth perspective, our data indicate that it is not metabolically inert regarding Fe homeostasis. The Mn-Fe antagonism becomes progressively more pronounced at higher supplementation levels (40 and 80 mg/kg). Consequently, a supplementation level at or below 20 mg/kg is recommended to minimize the risk of impairing Fe status in weaned pigs fed a corn–soybean diet. In addition, while the robust experimental design and consistent dose-dependent responses across 128 weaned pigs provide confidence in these findings, further studies with larger herd sizes are warranted to confirm these results and explore more subtle effects. Additional research is also needed to fully establish the physiological relevance of *DMT1* and *FPN1* to Mn absorption and the effects of high dietary Mn on the metabolism of other divalent cations.

## Figures and Tables

**Table 1:** Ingredients and nutritional composition of the experimental basal diet †.

Ingredient	Content, %
Corn	56.00
Soybean meal	28.00
Soybean oil	2.50
Whey, spray	5.50
Fish meal	5.00
Dicalcium phosphate	0.60
Limestone	1.00
Sodium chloride	0.40
Vitamin-mineral premix ‡	1.00
Total	100.00
Nutritional composition	
DE, MJ/kg	14.30
CP, %	20.90
CF, %	1.88
Calcium, %	0.89
Total phosphorus, %	0.65
Lysine, %	1.19
Methionine, %	0.36
Threonine, %	0.82
Mn §, mg/kg	39.22
Fe, mg/kg	125.00

† Control diet; no supplemental Mn; ‡ Supplied per kilogram of diet: VA 6300 IU, thiamine 3.50 mg, riboflavin 12.25 mg, pyridoxine 5.25 mg, cobalamin 0.05 mg, cholecalciferol 700 IU, vitamin E 28.00 IU, vitamin K3 5.00 mg, pantothenic acid 35.00 mg, Niacin 52.50 mg, chlorine chloride 400 mg, biotin 0.08 mg, folic acid 1.05 mg, Fe (as ferrous sulfate) 100.00 mg, Cu (as copper sulfate) 9.00 mg, Zn (as zinc sulfate) 100.00 mg, I (as potassium iodide) 0.60 mg, and Se (as sodium selenite) 0.30 mg; DE, digestible energy; CP, crude protein; CF, crude fiber; § The Mn and Fe content of diets was analyzed using flame atomic absorption spectrometry.

**Table 2:** RT-PCR primer sequences.

Gene	Accession Number	Primer Sequence (5′-3′)	Product Size (bp)	Exon Junction
*DMT1*	AM183784	F: CGAGAAGATCGCCATTCCTG	149	Exon 3-5
		R: ATCCAGCCACTGCTCCAGAC		
*FPN1*	AK397115	F: CGTCACTGGTCATCCAGAATGT	110	Exon 4-5
		R: AGTAAGAACCCATCCATGGTACA		
*GAPDH*	NM_001206359	F: TGTGTTCCGTGCATTGCCAG	70	Exon 1-3
		R: CGGCCAAATCCGTTCACTCC		

*DMT1*, divalent metal transporter 1; *FPN1*, ferroportin 1; *GAPDH*, glyceraldehyde-3-phosphate dehydrogenase.

**Table 3:** Effects of dietary Mn supplementation on growth performance in weaned pigs.

Item	Supplemental Mn, mg/kg of Diet †				SEM	*p*-Value ‡	
	0	20	40	80		Linear	Quad
ADFI	720.47	728.58	732.68	730.39	9.72	0.825	0.626
ADG	410.00	437.50	435.09	421.96	10.10	0.243	0.155
FE	1.76	1.67	1.69	1.73	0.05	0.534	0.397

† Data are least squares means (*n* = 4 per treatment); ‡ Linear and quadratic effects of increasing Mn concentration; ADFI reported on an as-fed basis; ADFI, average daily feed intake; ADG, average daily gain; FE, feed efficiency = ADFI/ADG; SEM, standard error of the mean.

**Table 4:** Effects of dietary Mn supplementation on hematological status in weaned pigs.

Item	Supplemental Mn, mg/kg of Diet †				SEM	*p*-Value ‡	
	0	20	40	80		Linear	Quad
Blood							
Hemoglobin, g/L	112.75 ^a^	117.50 ^a^	110.00 ^ab^	95.25 ^b^	5.56	0. 019	0.033
Hematocrit, %	42.18	43.50	42.30	43.23	1.29	0.707	0.932
Serum							
Fe, μg/mL	2.16 ^a^	2.18 ^a^	2.04 ^ab^	1.79 ^b^	0.10	0.008	0.024
Mn, μg/mL	0.115 ^b^	0.130 ^ab^	0.138 ^a^	0.143 ^a^	0.001	0.008	0.014

^a,b^ Means with different letters within a line differ significantly (*p* < 0.05). † Data are least squares means (*n* = 4 per treatment); ‡ Linear and quadratic effects of increasing Mn concentration; SEM, standard error of the mean.

**Table 5:** Effects of dietary Mn supplementation on tissue Mn and Fe content (µg/g tissue) in weaned pigs.

Item	Supplemental Mn, mg/kg of Diet †				SEM	*p*-Value ‡	
	0	20	40	80		Linear	Quad
Mn							
Liver	1.87 ^b^	2.82 ^a^	3.03 ^a^	3.07 ^a^	0.09	<0.001	<0.001
Kidney	1.26 ^b^	1.39 ^ab^	1.44 ^a^	1.46 ^a^	0.06	0.030	0.032
Pancreas	1.40 ^b^	1.47 ^ab^	1.48 ^ab^	1.55 ^b^	0.04	0.017	0.060
Heart	1.01 ^b^	1.64 ^ab^	1.95 ^a^	2.17 ^a^	0.24	0.004	0.008
Bone	1.89 ^b^	2.42 ^a^	2.46 ^a^	2.48 ^a^	0.05	0.004	<0.001
Fe							
Liver	90.86 ^b^	89.79 ^ab^	81.27 ^a^	77.60 ^a^	3.26	0.005	0.021
Kidney	41.06	39.97	39.90	40.66	1.67	0.935	0.843
Pancreas	18.18	18.25	18.08	18.29	1.35	0.997	0.998
Heart	58.79	58.59	62.36	70.44	6.35	0.142	0.328
Bone	14.82 ^a^	12.08 ^b^	10.60 ^c^	9.97 ^c^	0.47	<0.001	<0.001

^a,b,c^ Means with different letters within a line differ significantly (*p* <0.05). † Data are least squares means; ‡ Linear and quadratic effects of increasing Mn concentration; SEM, standard error of the mean.

**Table 6:** Effects of dietary Mn on apparent digestibility of Mn and Fe.

Item	Supplemental Mn, mg/kg of Diet †				SEM	*p*-Value ‡	
	0	20	40	80		Linear	Quad
Mn intake (mg/d)	24.66 ^c^	38.04 ^b^	50.44 ^a^	76.71 ^a^	0.47	<0.001	<0.001
Fecal Mn (mg/d)	16.17 ^c^	19.88 ^b^	29.55 ^b^	47.41 ^a^	1.42	<0.001	<0.001
Mn digestibility (%)	34.43 ^b^	47.74 ^a^	41.42 ^b^	38.20 ^b^	0.03	0.030	0.032
Fe intake (mg/d)	204.44	208.85	207.04	209.22	2.19	0.198	0.401
Fecal Fe (mg/d)	150.75 ^b^	155.90 ^ab^	165.00 ^ab^	167.75 ^a^	4.88	0.007	0.025
Fe digestibility (%)	26.26 ^a^	25.35 ^ab^	20.31 ^ab^	19.82 ^b^	1.97	0.017	0.060

^a,b,c^ Means with different letters within a line differ significantly (*p <* 0.05). † Data are least squares means; ‡ Linear and quadratic effects of increasing Mn concentration; SEM, standard error of the mean.

## Data Availability

The original contributions presented in this study are included in the article. Further inquiries can be directed to the corresponding author.
